# Circulating and Exosomal microRNA-33 in Childhood Obesity

**DOI:** 10.3390/biomedicines11082295

**Published:** 2023-08-18

**Authors:** Manuela Cabiati, Letizia Guiducci, Emioli Randazzo, Valentina Casieri, Giovanni Federico, Silvia Del Ry

**Affiliations:** 1 Laboratory of Biochemistry and Molecular Biology, Institute of Clinical Physiology, CNR, 56124 Pisa, Italy; manuela.cabiati@cnr.it (M.C.); letizia.guiducci@cnr.it (L.G.); 2 Unit of Pediatric Endocrinology and Diabetes, Department of Clinical and Experimental Medicine, University of Pisa, 56124 Pisa, Italy; emioli.randazzo@gmail.com (E.R.); giovanni.federico@med.unipi.it (G.F.); 3 Unit of Translational Critical Care Medicine, Scuola Superiore Sant’Anna, 56126 Pisa, Italy; valentina.casieri@santannapisa.it

**Keywords:** microRNA-33, obesity, childhood, Real-Time PCR

## Abstract

Background: MicroRNA-33 may control a wide range of different metabolic functions. Methods: This study aims to assess the miR-33a circulating profile in normal-weight (N = 20) and obese (O = 30) adolescents and to correlate its expression levels to their metabolic parameters. In a subset of subjects, we compared circulating miR-33a with exosomal miR-33a. Results: Metabolic parameters were altered in O, with initial hyperinsulinemia. Circulating miR-33a was significantly higher in O than in N (*p* = 0.0002). Significant correlations between miR-33a and auxological and metabolic indices (Insulin *p* = 0.01; Cholesterol *p* = 0.01; LDL *p* = 0.01; HbA1c *p* = 0.01) were found. Splitting our population (O + N) into two groups, according to the median value of mRNA expression miR-33a levels (0.701), irrespective of the presence or absence of obesity, we observed that those having a higher expression of miR-33a were more frequently obese (87.5% vs. 12.5%; *p* < 0.0001) and had significantly increased values of auxological and metabolic parameters. Exosomes extracted from plasma of N and O carried miR-33a, and its expression was lower in O (*p* = 0.026). No correlations with metabolic parameters were observed. Conclusion: While exosome miR-33a does not provide any advantage, circulating miR-33a can provide important indications in an initial phase of metabolic dysfunction, stratifying obese adolescents at higher cardiometabolic risk.

## 1. Introduction

Metabolic syndrome (MetS), a condition strongly related to a greater risk of developing cardiovascular disease and type 2 diabetes mellitus [1], is characterized by the clustering of clinical conditions including central obesity, dyslipidemia, hypertension, and insulin resistance [1]. MetS is considered a major health problem, and recently, it has become increasingly relevant due to the exponential worldwide increase of obesity and the linked important therapeutic challenges [1,2]. Lifestyle variation, a healthy diet, and pharmacological treatment are suggested for managing this syndrome [3]; nevertheless, the mechanisms participating in metabolic dysregulation remain unclear and are intensively examined to find new therapeutic avenues. 

Many studies have focused their attention on the roles of metabolic enzymes; hormones; signaling molecules; and regulatory proteins, such as transcription factors and co-factors, in metabolic diseases. Recently, an important role as modulators of metabolic homeostasis has been attributed to RNAs, i.e., microRNAs (miRNAs) [4,5]. MicroRNAs are a cluster of short, non-coding RNAs able to influence gene expression by suppressing their translation or by promoting the degradation of mRNAs [6]. MicroRNAs are among the most plentiful gene regulators in humans and have now been linked to many physiological and pathological processes, including obesity and diabetes [7,8,9]. 

The increasing information about miRNAs and their molecular actions enabled innovative engineering and applications of these biomarkers, particularly in medical therapy [10]. The use of miRNAs in medicine as new biomarkers could be facilitated by the fact that they may be carried by exosomes, which are small membrane vesicles of 40–100 nm in size [10].

The most plentiful miRNA in lipoprotein particles, generally suggested as an important regulator of lipid metabolism, is miR-33 [7,11,12]. The family of miR-33 consists of miR-33a and miR-33b, which are located in intron 16 of human SREBP-2 and SREBP-1 genes (master regulators of genes involved in cholesterol/lipid biosynthesis and trafficking), respectively [13]. MiR-33a directly targets the cholesterol transporters Abca1 and Abcg1, which are responsible for the efflux of cholesterol from the cell, underling the importance of this miRNA in cholesterol metabolism. MiR-33b is also implicated in fatty acid β-oxidation, as carnitine palmitoyltransferase (Cpt1a) [14]. It seems that metabolic regulation by miR-33 is more complex than previously known. Many studies reported an increase in Abca1 expression and plasma HDL levels [11,14,15,16]. 

A possible deleterious metabolic consequence of antagonizing miR-33 was shown by some studies in knockout mice [17,18]. Their results suggested that these metabolic abnormalities might be due to elevated SREBP-1 protein in the liver (SREBP-1 was shown to be a target of miR-33); in agreement, it was also reported that expression of SREBP-1 lipogenic target genes, such as FASN and ACC1, is elevated in the liver of mice treated regularly with miR-33-targeting antisense oligonucleotides [19]. In addition, it was found that loss of miR-33 leads to reduced insulin sensitivity in the liver, white adipose tissue, and skeletal muscle, even in mice fed on a chow diet [18]. Hyperinsulinemic–euglycemic clamp studies discovered that miR-33 knockout mice exhibited reduced glucose uptake in white adipose tissue and skeletal muscle, reduced suppression of endogenous glucose production in the liver, and reduced capacity to suppress the release of non-esterified fatty acids from white adipose tissue [18]. In miR-33 null mice fed on a high-fat diet, it was observed an increased infiltration of inflammatory cells in white adipose tissue, suggesting that complete miR-33 loss, especially in the context of a high-fat diet, may have significant harmful metabolic consequences that are comparable to obesity-associated insulin resistance, type 2 diabetes, and white adipose tissue inflammation in humans.

Thus, the loss of miR-33 promotes insulin resistance in key metabolic tissues [18]. MiR-33, in fact, targets genes involved in many important metabolic functions, including fatty acid and lipid metabolism, insulin signaling, and mitochondrial function [20,21,22,23,24]. The potential of miR-33 to control many different metabolic functions hints at its involvement in regulating diverse metabolic functions in many tissues. This is especially interesting if one considers that miR-33 is encoded within intronic regions of the SREBP-2 and SREBP-1 genes, which are among the most important regulators of cellular cholesterol and fatty acid metabolism, and that it has been demonstrated to be differentially regulated in many different metabolic tissues under conditions of obesity and insulin resistance [25]. Taken together, these studies clearly indicated that miR-33 is a central player in the regulation of liver metabolism, suggesting that it might have a potential role in the treatment of metabolic diseases. Since being obese in childhood increases the risk of being obese as an adult [26,27], evaluating the expression levels of miR-33 in obese children could provide important indications in those at higher risk of developing metabolic complications, such as dyslipidemia, hypertension, and impaired glucose metabolism, later in life [28]. So, the primary aim of the present investigation was to evaluate the miR-33 circulating profile in normal-weight (N) and obese (O) adolescents and to correlate its expression levels with metabolic parameters to confirm its involvement in metabolic complications. In addition, bearing in mind the possible use of miRNA in medical therapy, together with exosomes as miRNA carriers [10], we reasoned that the evaluation of miRNA-33 expression in exosomes could be helpful in the screening and surveillance of metabolic complications in obese adolescents, allowing earlier and more efficient prevention, diagnosis, and treatment. Considering this and our recent observation of the presence of miRNA-33a in the exosomes of obese adolescents [29], we established, as a secondary aim of the present study, comparing the expression levels of circulating miRNA-33a with those found in exosomal vesicles in a subset of our cohort, looking at possible correlations between miRNA-33a expression in exosomes and some metabolic indices.

## 2. Materials and Methods

### 2.1. Subjects and Plasma Collection 

We enrolled 50 adolescents, 30 obese (O) and 20 age- and sex-matched normal-weight (N) subjects, as a control group. Table 1 summarizes the clinical characteristics and body composition of the population participating in the study. Obese adolescents were referred as outpatients to the Unit of Pediatric Endocrinology and Diabetes, Department of Clinical and Experimental Medicine, University of Pisa, Italy. They had primary obesity, not induced by drug or diseases, and they were not affected by diabetes or cardiac dysfunction. Obesity was defined according to the criteria of the International Task Force on Obesity in childhood, using population reference data specific for age and sex for body mass index (BMI) [30]. As also reported in our previous papers [29,31,32,33,34,35,36], the normal-weight adolescents were healthy subjects, who repeated a blood examination after an intervening disease. At the time of blood sampling, they had not taken drugs for at least one week, and their blood results, including indices of inflammation, were in the normal range. BMI was calculated using the formula weight [(Kg)/height (m)^2^] [30]. We used the same National reference data [30] to calculate the BMI z-score and Height z-score. Sexual development was assessed according to Tanner’s stages [37,38]. Total body fat (%) was measured using the Tanita BC-418 Segmental Body Composition Analyzer (Tanita Corporation, Tokyo, Japan) [39]. Blood pressure was measured by trained investigators, according to a standardized protocol [40]. 

We collected blood samples from all the subjects by venipuncture, performed in the morning after overnight fasting. To evaluate the circulating levels of metabolic parameters, blood samples were collected into ethylenediaminetetraacetic acid (EDTA) (1 mg/mL) and lithium-heparin-containing vials [29,31,32,33,34,35,36]. They were measured by appropriate commercial kits, as previously reported [29,31,32,33,34,35,36]. 

The investigation conforms to principles outlined in the Declaration of Helsinki [41]. The study was approved by the local Ethics Committee, and informed consent was obtained from the parents of each subject.

### 2.2. Circulating miRNA-33a Extraction and Reverse Transcription 

As previously reported [31,32], the extraction of miRNAs was conducted by using the miRNeasy Serum/Plasma Kit (Qiagen S.p.a., Milano, Italy). Briefly, 200 μL of plasma was lysed in an adequate lysis reagent and applied to silica-membrane columns. To monitor RNA recovery and reverse transcription efficiency, a Spike-In Control (C. elegans miR-39 miRNA mimic) was used as the internal control for plasma miRNA expression profiling. High-quality RNA was then eluted in a small volume (14 μL) of RNase-free water; samples were stored at −80 °C after integrity, purity, and concentration evaluation. The fraction of mature miRNAs was reverse-transcribed using the miScript II RT kit (Qiagen S.p.a., Milano, Italy), starting from a 1 μg/sample in 20 μL of final reaction volume (60 min at 37 °C, 5 min at 95 °C, and 4 °C for ∞). cDNA samples were stored, as appropriate, and diluted at +4 °C.

### 2.3. Exosome Isolation and Characterization, Vesicular RNA Extraction and Reverse Transcription

In a subset of the study population (Ssp, *n* = 23), we carried out exosome isolation. Table 2 summarizes the clinical characteristics of the subjects included in the subgroups. 

As reported in a previous study of ours [29], exosomes were extracted from 600 μL of plasma using a dedicated and innovative assay (exoRNeasy mini/midi kit, QIAGEN GmbH, Hilden, Germany); before RNA extraction and in a random manner, we analyzed the morphology of exosomes with Transmission Electron Microscopy (TEM). The presence of specific exosomal proteins in our preparations was evaluated by Western blotting analysis. In particular, we used primary antibodies to detect human CD9 (monoclonal antibody, 1:1000, #SA35-08, Novus Biological, San Diego, CO, USA), human TGS101 (polyclonal antibody, 1:1000; #T5701, Sigma-Aldrich Chemical, St. Louis, MO, USA), and human Alix (monoclonal antibody, 1:1000, #2171, Cell Signaling Technology, Boston, MA, USA) as established exosomal markers, while human Calnexin (polyclonal antibody, 1:1000, ab10286, Abcam, Cambridge, UK) was used as a negative exosomal marker. Densitometric analysis of protein bands was carried out with Image J software 1.52t (National Institutes of Health, Bethesda, MD, USA). Briefly, for exosome RNA extraction, plasma samples were prefiltered using syringe filters excluding particles larger than 0.8 µm (Millipore^®^ Membrane Filter, 0.8 µm pore size, MILLEX-AA, Merck, D); then, exosomes were isolated, adding in a 1:1 ratio of a 2× binding buffer (XBP). Next, samples were transferred into the ExoEasy membrane affinity column, to bind the exosomes to the membrane, and then centrifuged at 500× *g* for 2 min at RT. After discarding the flow-through, 3.5 mL of wash buffer (XWP) was added to the column, according to the starting plasma quantity, and centrifuged at 5000× *g* for 5 min at RT. Vesicles were lysed by adding to the spin column a phenol/guanidine-based combined lysis reagent (QIAzol), and the lysate was collected by serial centrifugations. After the lysis, elution step, and addition of chloroform, the resulting samples were separated into aqueous and organic phases by centrifugation. Ethanol was added to the aqueous phase, and samples were applied to the RNeasy MinElute spin column, washed, centrifuged, and finally eluted in RNase-free water (16 μL). The samples were then stored at −80 °C; RNA integrity, purity, and concentration were assessed by measuring absorbance at 230, 260, and 280 nm (NanoDrop Thermofisher, Waltham, MA, USA) and applying the Beer–Lambert law (expected values between 1.8 and 2.1, for protein contamination). The exosomal miRNA reverse transcription was carried out with the same procedure (miScript ^®^ II RT kit, Qiagen, Hilden, Germany) used for circulating miRNA-33a retro-transcription.

### 2.4. Real-Time PCR Analysis for Circulating and Exosomal miRNA-33a

Real-time PCR reactions were carried out in duplicate using a Bio-Rad C1000™ thermal cycler system (CFX-96 Real-Time detection system, Bio-Rad). To monitor cDNA amplification, a fluorogenic DNA binding dye, EvaGreen (SsoFAST EvaGreen Supermix, Bio-Rad Laboratories Inc., Hercules, CA, USA), was used. 

The miR-33a primer sequence was synthesized by Sigma Aldrich (Milan, Italy). In particular, the GenBank accession number for hsa-miR-33a was NR_029507, and the forward primer sequence (5′—3′) was CAATGTTTCCACAGTGCATCAC. The Ce_miR-39 (miRNA mimic Ce_miR-39_1 miScript Primer Assay) was employed to normalize miRNA data. All experiments were carried out according to the MIQE (Minimum Information for publication of Quantitative Real-Time PCR Experiments) guidelines [42].

### 2.5. Statistics

Statistical analysis was performed using Statview 5.0.1 Software for Windows (SAS Institute, Inc., Cary, NC, USA). Relative quantification was performed by the ΔΔCt method using Bio-Rad’s CFX96 manager software v.3.1 (CFX-96 Real-Time PCR detection systems, Bio-Rad Laboratories Inc., Hercules, CA, USA). Skewed variables were log-transformed before statistical analysis. Differences between more than two independent groups were analyzed by Fisher’s test after ANOVA, and relations between variables were assessed by linear regression analysis. The results were expressed as mean ± S.E.M., and a *p*-value < 0.05 was considered significant.

## 3. Results

### 3.1. Clinical Characteristics

**Figure 1:**
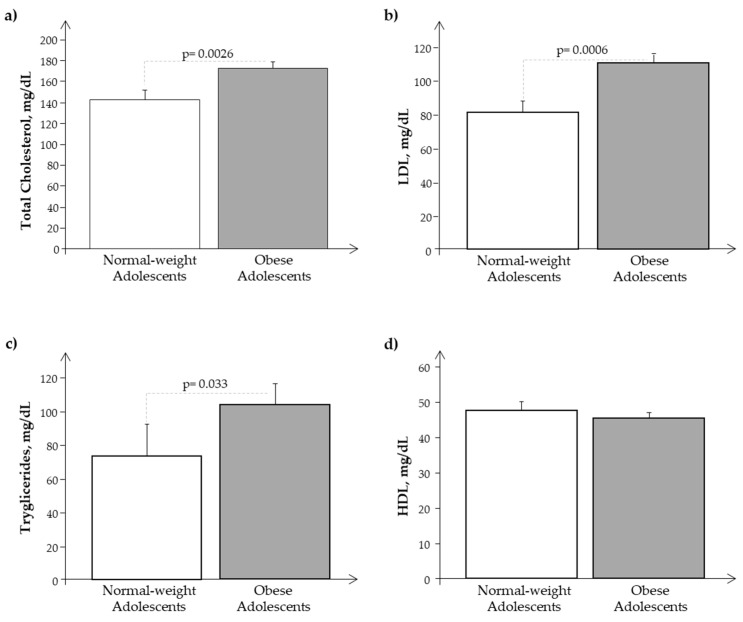
Plasma levels of (**a**) cholesterol, (**b**) LDL, (**c**) triglycerides, and (**d**) HDL in obese (O) with respect to normal-weight (N) adolescents.

As reported in Table 1, obese subjects showed significant differences as regards weight, BMI, BMI z-score, fat mass, HOMA-IR, and WC with respect to normal-weight subjects, and we observed the same in the subset of adolescents in whom we analyzed isolated exosomes (Table 2). Obese adolescents presented also a significantly slight increase in pubertal development as a result of their nutritional excess. Systolic and diastolic blood pressure results were similar in N and O. Moreover, as reported in Figure 1a–c, O showed higher significant levels of cholesterol, LDL, and triglycerides, while HDL values were similar between the two groups (Figure 1d). 

**Figure 2:**
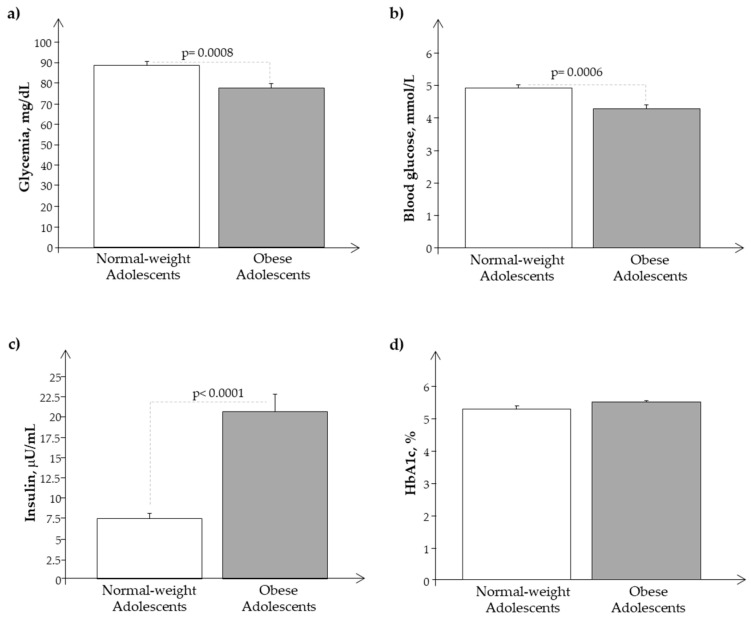
Plasma levels of (**a**) glycemia, (**b**) glucose, (**c**) insulin, and (**d**) Hb1ac in obese (O) with respect to normal-weight (N) adolescents.

We observed a significant reduction of glucose plasma levels in O with respect to N in the presence of higher plasma levels of insulin and Hb1ac, confirming a condition of hyperinsulinemia in obese subjects (Figure 2a–d). 

**Figure 3:**
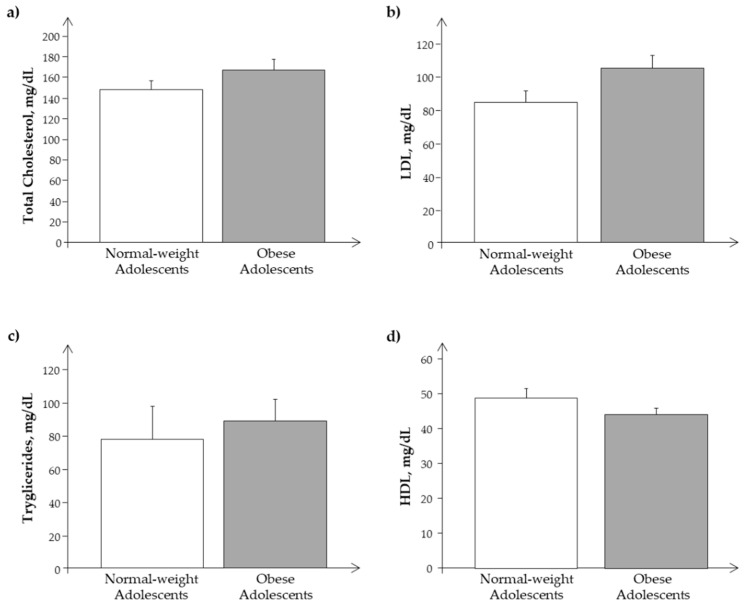
Plasma levels of (**a**) cholesterol, (**b**) LDL, (**c**) triglycerides, and (**d**) HDL in the subset of the study population, Ssp. (obese and normal-weight adolescents).

**Figure 4:**
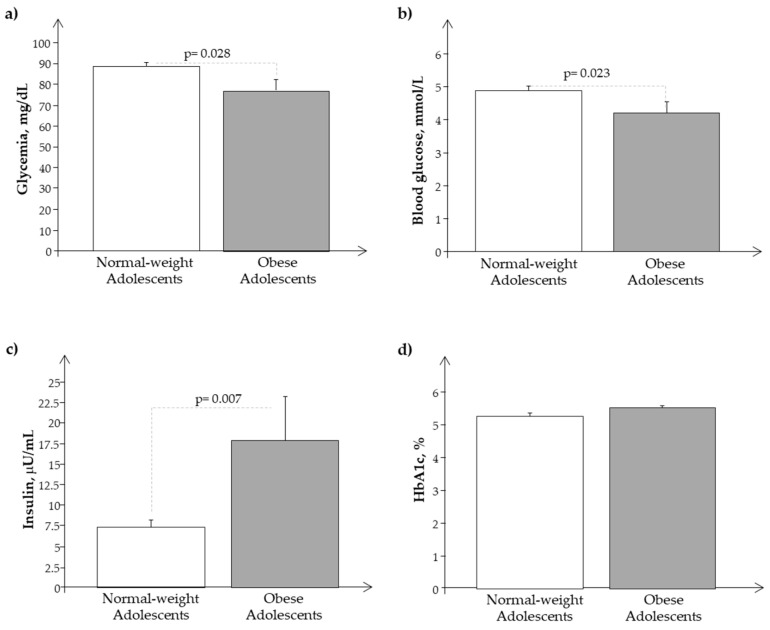
Plasma levels of (**a**) glycemia, (**b**) glucose, (**c**) insulin, and (**d**) Hb1ac in the subset of the study population, Ssp. (obese and normal-weight adolescents).

We observed a similar behavior of the same parameters and analytes in the Ssp population, as reported in Figure 3 and Figure 4. 

### 3.2. miRNA-33a Expression in Plasma and Exosome Samples 

**Figure 5:**
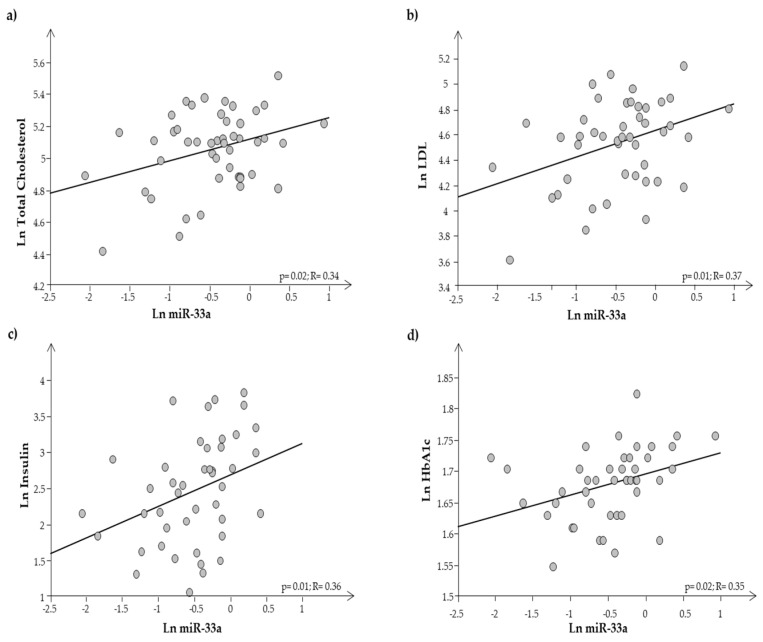
Regression analysis between miR-33a and (**a**) total cholesterol, (**b**) LDL cholesterol, (**c**) insulin, and (**d**) HbA1c.

Plasma expression of miR-33a resulted in significantly higher expression in O than in N (0.9 ± 0.08 vs. 0.5 ± 0.07, respectively; *p* = 0.0002). It was interesting to note that miR-33a was significantly related to both auxological parameters and metabolic indices, such as cholesterol, LDL, insulin, and HbA1c (Figure 5a–d). 

After calculating the miR-33a expression levels in plasma samples in the entire population (O + N) as the median value, we used it as the cut-off (0.701) to classify our population into two groups of subjects, irrespective of the presence or absence of obesity: those having lower miR-33a expression levels (Group 1: <median value) and those with higher miR-33a expression levels (Group 2: ≥ median value). Subsequently, comparing some clinical and metabolic data between Group 1 (*n* = 23) and Group 2 (*n* = 24), we observed that those having higher miR-33a expression levels (Group 2) were more frequently obese (Group 2 vs. Group 1 = 87.5% vs. 12.5%; *p* < 0.0001) and had an increased BMI z-score (Group 2 vs. Group 1 = 2.5 ± 0.21 vs. 1.5 ± 0.2; *p* = 0.0013) and fat mass (Group 2 vs. Group 1 = 35.3 ± 1.9 vs. 21.5 ± 2.0%; *p* < 0.0001). 

In comparison with Group 1, Group 2 had significantly higher levels of circulating insulin (Group 2 vs. Group 1 = 20.3 ± 2.5 vs. 10.4 ± 1.7 µU/mL; *p* = 0.0005) and HbA1c (Group 2 vs. Group 1 = 5.5 ± 0.057 vs. 5.2 ± 0.05%, *p* = 0.0004 corresponding to 37.0 ± 0.61 vs. 33.7 ± 0.64 mmol/mol, *p* = 0.0005). Also, plasma levels of total cholesterol (Group 2 vs. Group 1 = 169.7 ± 6.6 vs. 152.8 ± 7.9 mg/dL), LDL cholesterol (Group 2 vs. Group 1 = 105.9 ± 6.3 vs. 90.7 ± 6.4 mg/dL), HDL cholesterol (Group 2 vs. Group 1 = 46.6 ± 1.9 vs. 44.4 ± 2.1 mg/dL), and triglycerides (Group 2 vs. Group 1 = 95.5 ± 12.4 vs. 87.6 ± 17.3 mg/dL) resulted in being higher, even if not significantly, in Group 2 than in Group 1, while blood glucose showed an opposite behavior, being lower in Group 2 than in Group 1 (4.34 ± 0.136 vs. 4.7 ± 1.33 mmol/L, *p* = 0.045 corresponding to 78.47 ± 2.3 vs. 85.3 ± 2.29 mg/dL, *p* = 0.044).

We performed the same evaluations in exosome samples obtained from the subjects of the Ssp.

We previously reported that, following the MISEV 2018 guideline [43], TEM analysis revealed that the exoRNeasy mini/midi kit procedure isolated round-shaped intact vesicles from pre-filtered plasma samples and that Western blotting analysis, detecting proteins specifically expressed in exosomes, confirmed TEM results [29].

Exosomes extracted from plasma of both normal-weight and obese subjects carried miR-33a, but its expression levels were lower in exosomes obtained from O than from N (0.58 ± 0.29 vs. 1.33 ± 0.29, respectively; *p* = 0.026), confirming our previous results in obese adolescents [29]. We also observed some significant correlations between the expression of exosomal miR-33a and auxologic parameters (BMI: r = 0.49, *p* = 0.01; fat mass: r = 0.48, *p* = 0.04; HOMA: r = 0.42, *p* = 0.04), but not with metabolic indices. By dividing Ssp into two groups, according to the median value of miR-33a expression levels (1.085), irrespective of the presence or absence of obesity, we did not obtain any additional information.

**Figure 6:**
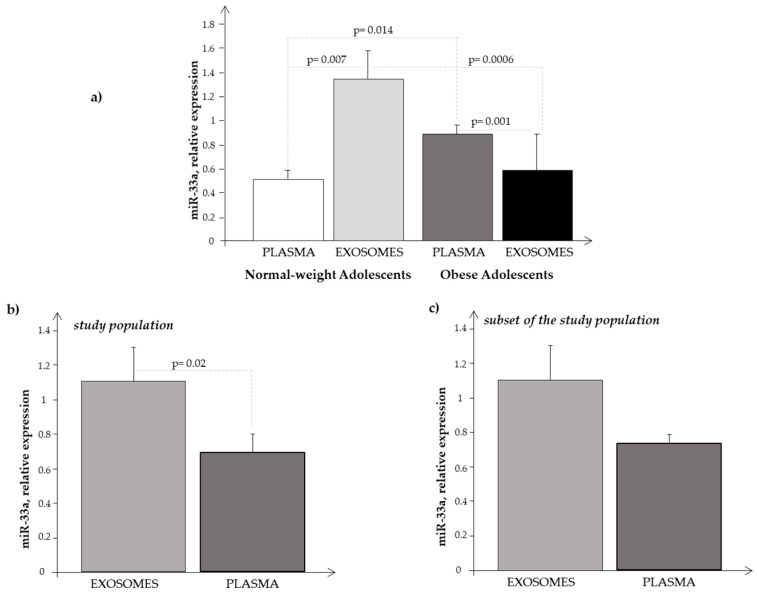
Expression level of miR-33a obtained in (**a**) plasma and in exosome samples in normal-weight (light-grey bar) and obese (black bar) subjects, respectively; (**b**) in plasma (light-grey bar) and in exosome (black bar) samples considering the subjects as a whole (N + O); and (**c**) in plasma (light-grey bar) and in exosome (black bar) samples considering the subjects of the Ssp as a whole (N + O).

Comparing the results obtained in plasma and in exosome samples (Figure 6a), we observed that miR-33a showed higher expression levels in exosomes than in plasma samples of normal-weight subjects (*p* = 0.0073), while in obese subjects, we noted the opposite, that is, an increased expression in plasma samples rather than in exosomes (*p* = 0.001).

We also compared the expression levels of circulating miR-33a with that of exosomal miR-33a observed in the whole population (O plus N subjects), and we found that the levels were significantly higher in exosomes than in plasma samples (Figure 6b). Performing the same analysis in the subjects of the Ssp, we observed the same trend (Figure 6c), even if the differences were not significant, probably due to the smaller number of subjects analyzed, confirming somehow a greater expression of miR-33a at the exosomal level.

Finally, we observed a significant negative correlation between circulating and exosomal miR-33a expression levels (r = −0.65, *p* = 0.001).

## 4. Discussion

Our obese adolescents showed a metabolic disorder, including a certain degree of hyperinsulinemia. Data from the literature reported that miR-33a has an important role in obesity, diabetes, and metabolic syndrome [7,8,9,44,45,46]. In the present study, we analyzed the circulating levels of this miRNA in a group of obese adolescents not having yet a clear metabolic syndrome, but only some metabolic alterations of the syndrome. Interestingly, they already showed a significant increase in the circulating expression levels of miR-33a as compared to N. In particular, we observed the presence of a significant direct correlation between the expression level of circulating miR-33a and total cholesterol, LDL cholesterol, insulin, and HbA1c in our cohort, suggesting the role of this biomarker in lipid regulation during adolescence. Thus, our results not only confirmed some observations previously reported in animal and human studies [7,8,9,44,45,46], but also, they highlighted the potential of the circulating expression of this miRNA as an early biomarker useful to discriminate subjects at higher metabolic risk. The use of an early marker in childhood could help in the early recognition of obese children/adolescents at higher risk of metabolic alterations that may lead to cardiometabolic diseases in adult life as a result of obesity in children/adolescents. Expression levels of circulating miR-33a correlated significantly with metabolic parameters, in addition to the auxological ones, and this was also observed after dividing the whole population (obese plus normal-weight subjects) into two groups, according to the median value of miR-33a expression levels. Despite plasma miRNAs and exosome miRNAs having been extensively studied as diagnostic or therapeutic biomarkers in several disease-related research studies, few studies determined whether miRNA expression levels were different between plasma and plasma-derived exosomes. This was the reason why we decided to compare the expression level of circulating miR-33a with that of exosomal vesicles obtained from the same subjects (considering them as a whole, O + N), and we found that miR-33a was mostly transported by exosomes (Figure 6b,c). Analyzing the same results separately in normal-weight and in obese subjects, we observed the same trend in N, while the opposite was seen in O, that is, miR-33a expression levels were higher in plasma samples than in circulating exosomes (Figure 6a). 

It is rather hard to explain why circulating and exosome miRNA behave so differently; we found an inverse correlation between the expression levels of circulating and exosomal miR-33a. However, some considerations can be drawn, as we recently reported in a paper by our group [29]. It should be considered that the expression of circulating and exosome miRNAs represents the result of their regulation in two diverse compartments: plasma and cell-derived exosomes. The tendency of miRNA in plasma summarizes, in a nonspecific manner, biological mechanisms originating from several districts (cells, tissues, etc.), while the expression of miRNA in the exosome may mirror the result of a more sophisticated regulation [47]. It is known the special role that exosomes and their cargo of non-coding RNAs (primary miRNAs) play in intercellular communication [48]. Moreover, exosome biogenesis and secretion are influenced by the metabolic status of the cell, which, in turn, depends on factors such as ceramide metabolism, endoplasmic reticulum stress, autophagy, and intracellular calcium [49]. After their release, exosomes interplay with their target cells by transferring their bioactive cargo into them. As a result, this transfer triggers phenotypic changes in the recipient cells [50]. Furthermore, since exosome-mediated intra-adipose and inter-organ communication is of great significance for energy metabolism, one alternative explanation could be that this special function of exosomes in adipose tissue biology may be interrupted in obesity [50]. These reflections may help to explain, at least in part, the different results we found in examining the expression behavior of miR-33a in plasma and in exosome samples.

In the literature are well-described changes of miRNA patterns in biofluids in some diseases [51,52,53], including obesity, and our results on the expression of miR-33a in circulating exosomal vesicles seem to show that this evaluation does not provide additional indications on the cardiometabolic risk of our obese adolescents in adulthood.

The contribution of microRNA in glucose and lipid metabolism can provide solid indications in support of their role as key players in the regulation of complex metabolic pathways.

## 5. Conclusions

In adolescents, excessive intake of calories and following unbalanced diets too rich in sugars and saturated fats, in combination with stress and low levels of physical activity, can cause overweight, obesity, and metabolic alterations with a higher risk of developing cardiometabolic diseases in adult life.

The availability of a circulating biomarker, such as miR-33a, for early identification of obese adolescents at higher cardiometabolic risk provides an important tool for designing interventions to correct unhealthy lifestyles when these metabolic alterations are still reversible.

Finally, even if much remains to be understood about the role of miR-33 in conditions such as obesity, it may represent an ideal target for future therapies.

## Figures and Tables

**Table 1:** Demography, clinical characteristics, and body composition of study population.

	Normal-Weight Subjects	Obese Subjects	*p*
Age (years)	13.1 ± 0.16	12.2 ± 0.36	ns
Male:Female	9:11	18:12	ns
Pubertal Stage (Tanner score)	3.1 ± 0.18	3.6 ± 0.143	*0.0461*
Height (cm)	157.4 ± 0.96	155.4 ± 2.34	ns
Height z-score	0.22 ± 0.06	0.91 ± 0.2	*0.0086*
Weight (Kg)	50.3 ± 0.79	72.7 ± 3.1	*<0.0001*
BMI	20.2 ± 0.20	29.8 ± 0.79	*<0.0001*
BMI z-score	0.68 ± 0.08	2.91 ± 0.2	*<0.0001*
WC (cm)	84.3 ± 2.1	92.1 ± 1.78	*0.0093*
Fat Mass (%)	19.7 ± 1.51	36.7 ± 1.78	*<0.0001*
SBP (mmHg)	112.5 ± 1.8	108.0 ± 2.2	ns
DBP (mmHg)	62.2 ± 1.4	63.8 ± 1.7	ns
HOMA-IR	0.95 ± 0.09	2.5 ± 0.24	*<0.0001*

Table Legend: All data are expressed as mean ± SEM. BMI: Body Mass Index; WC: Waist Circumference; SBP: Systolic Blood Pressure; DBP: Diastolic Blood Pressure; HOMA-IR: HOmeostatic Model Assessment of Insulin Resistance; ns: not significant.

**Table 2:** Demography, clinical characteristics, and body composition of Ssp.

	Normal-Weight Subjects	Obese Subjects	*p*
Age (years)	13.1 ± 0.2	12.7 ± 0.78	ns
Male:Female	8:8	5:2	ns
Pubertal Stage (Tanner score)	3.09 ± 0.21	3.15 ± 0.42	ns
Height (cm)	157.5 ± 0.96	156.6 ± 6.3	ns
Height z-score	0.24 ± 0.06	0.46 ± 0.4	*0.032*
Weight (Kg)	50.3 ± 0.70	74.9 ± 7.9	*<0.0001*
BMI	20.2 ± 0.20	30.2 ± 2.0	*<0.0001*
BMI z-score	0.68 ± 0.09	2.8 ± 0.23	*<0.0001*
WC (cm)	84.8 ± 2.3	89.8 ± 3.3	*0.0085*
Fat Mass (%)	19.3 ± 1.68	39.2 ± 10.8	*0.028*
SBP (mmHg)	113.9 ± 1.6	108.0 ± 4.5	ns
DBP (mmHg)	62.6 ± 1.5	58.8 ± 4.9	ns
HOMA-IR	0.95 ± 0.1	2.18 ± 0.53	*0.0036*

Table Legend: BMI: Body Mass Index; WC: Waist Circumference; SBP: Systolic Blood Pressure; DBP: Diastolic Blood Pressure; HOMA-IR: HOmeostatic Model Assessment of Insulin Resistance; ns: not significant.

## Data Availability

The data that support the findings of this study are available in IFC-CNR.
